# The Potential of Sequential Extraction in the Characterisation and Management of Wastes from Steel Processing: A Prospective Review

**DOI:** 10.3390/ijerph120911724

**Published:** 2015-09-18

**Authors:** Kiri J. Rodgers, Andrew Hursthouse, Simon Cuthbert

**Affiliations:** School of Science & Sport, University of the West of Scotland, Paisley Campus, Paisley PA1 2BE, UK; E-Mails: kiri.rodgers@uws.ac.uk (K.J.R.); Simon.Cuthbert@uws.ac.uk (S.C.)

**Keywords:** sequential extraction, chemical speciation, steel wastes, regulation

## Abstract

As waste management regulations become more stringent, yet demand for resources continues to increase, there is a pressing need for innovative management techniques and more sophisticated supporting analysis techniques. Sequential extraction (SE) analysis, a technique previously applied to soils and sediments, offers the potential to gain a better understanding of the composition of solid wastes. SE attempts to classify potentially toxic elements (PTEs) by their associations with phases or fractions in waste, with the aim of improving resource use and reducing negative environmental impacts. In this review we explain how SE can be applied to steel wastes. These present challenges due to differences in sample characteristics compared with materials to which SE has been traditionally applied, specifically chemical composition, particle size and pH buffering capacity, which are critical when identifying a suitable SE method. We highlight the importance of delineating iron-rich phases, and find that the commonly applied BCR (The community Bureau of reference) extraction method is problematic due to difficulties with zinc speciation (a critical steel waste constituent), hence a substantially modified SEP is necessary to deal with particular characteristics of steel wastes. Successful development of SE for steel wastes could have wider implications, e.g., for the sustainable management of fly ash and mining wastes.

## 1. Introduction

In 2013, approximately 1.6 billion tonnes of crude steel were produced worldwide [1,2] with as much as 400 kg of solid waste created per tonne of steel produced [3]. Steel wastes contain significant quantities of potentially toxic elements (PTEs) that require robust management, including assessment and disposal, to minimise environmental contamination. The European Union (EU) waste management protocols that govern approaches to assessment and disposal in member states [4] are based upon identifying the presence of hazardous substances and their potential impact on the environment and human health. This has resulted in the development of waste classification into inert, non-hazardous and hazardous classes, which are disposed of accordingly. These waste are disposed of accordingly with costs increasing annually throughout the majority of the EU causing financial pressures (not including any additional gate fees or operator charges [5,6,7,8]. This approach has been adopted by many European countries [7,9] leading to increased controls and higher costs for disposal, resulting in increased pressure to recycle and re-use materials within the industrial life cycle [10].

A better understanding of the chemical composition of the wastes generated during steel production can enhance their management, in particular for re-use, recycling or more suitable disposal options. This can only be viable if the methodology is robust and accessible for routine industrial application. The semi-continuous nature of steel manufacturing means that wastes, whilst broadly consistent in their primary characteristics, show high variability in concentration of many PTEs of high environmental concern. For metal contaminants, associations with reactive phases and chemical form (speciation) are fundamental controls on their release, transport and environmental impact. Speciation can be derived from direct characterisation techniques such as those using synchrotron x-ray techniques (e.g., XANES and EXAFS) [11] but compositional variability and the complexity of the analytical procedures makes them unattractive for routine analysis.

Fractionation methods have been applied widely to characterise the environmental reactivity of PTEs in fluid and solid phases for many years. For solid phases sequential extraction (SE) is one such technique that can be used to characterise different material fractions, each of which consists of phases with which PTEs are associated. Over the past four decades the majority of SE applications have focused on soil and sediment samples [12], but application to industrial wastes (including those from steel production) has been much less common. Adaption of SE methods to different materials requires that differences in their reactivity (e.g., buffering capacity) be taken into account. Modification of extraction protocols has been shown [13] to be prone to interference from sample specific solubility limiting reactions [14]. Nevertheless, SE leaching protocols developed for a variety of wastes from construction, mining and incineration operations have provided valuable information about their characteristics [13,15,16] that aids in waste management decision support.

In this contribution we explore how such an approach may make SE applicable as a characterisation tool for steel wastes. We review the development of sequential extraction procedures, identify options for future investigation, and highlight problematic components of waste streams. The goal for the successful development of improved characterisation methods is to provide a stepping-stone for the development of alternative end-of-life-cycle industrial waste management [17].

### 1.1. Need for Improvements to Screening Protocols?

**Figure 1:**
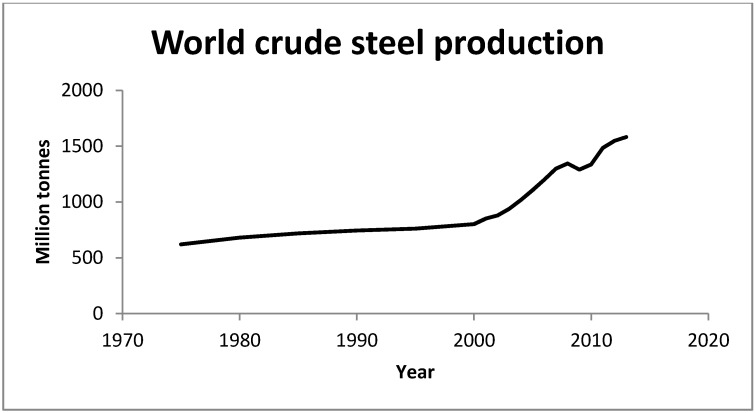
Crude steel produced between 1970–2013 (adapted from World steel [2]).

The steel industry is an important and highly relevant case study due to the continuing growth of steel production (Figure 1), world steel demand grew by 3.6% and was predicted to grow by 3.1% in 2014 [2,18,19] the remainder still represents a very large mass requiring disposal, e.g., the 1.6 billion tones of crude steel produced in 2013 as a worst case scenario generated up to 64 million tonnes of waste, so such growth has great significance for waste managment.

Increasing financial pressure on waste producers include annual increases in landfilling costs [19,20], more stringent regulatory thresholds, and increased financial penalties. These have been designed to reduce the attractiveness of landfill disposal options [7,8,19]. This has stimulated the need to investigate alternative solutions for sustainable resource use, to reduce the cost and environmental pollution, increase resource utilization and reduce waste generation. This is particularly the case with solid wastes due to the difficulty of characterizing them and identifying the hazardous fractions present that could be treated.

Screening tools that enable the producers and regulators of wastes to better understand the reactivity waste constituents would allow for effective (“smarter”) decision making, because it would provide more detailed information about the likely fate of wastes in the environment and the potential for resource recovery. Measurement of total PTE concentrations is commonly used as a basis for the prediction of environmental impact , but for many constituents environmental reactivity is poorly related to total content [12,20,21]. Thus total PTE analysis can be considered a “worst case scenario” approach and risks classifying relatively inert materials in higher risk categories, with a commensurate financial burden. Easily soluble contaminants are identified with leaching-based methods such as WAC testing (see below); however this does not provide environmentally realistic predictions. For example, commonly-used simulations do not even emulate natural rainfall, which is a mildly acidic solution, this is particularly the case for soils, however it was suggested that the use of synthetic acid rain as opposed to distilled water was not necessary for municipal wastes because of the because of the high alkalinity of the municipal solid wastes [22]. Longer term reactivity tests supplemented by buffering data may be useful, but can be less attractive due to operational complexity and time dependent feedback.

The ability to chemically speciate toxic components, wih regards to phase association, in wastes provides crucial information about the solid-phase or dissolved species hosts for PTEs. This allows for better prediction and understanding of an element’s environmental fate and potential impact [23]. However, precise speciation is often time-consuming or requires expensive analytical facilities, so sometimes the development of indirect proxies for characterisation is necessary. This is discussed further in Section 1.3 below.

### 1.2. Regulatory Testing

Waste Acceptance Criteria (WAC) is the compliance test implemented in July 2005 under the Landfill Regulations Amendment; England and Wales [24]; and Scotland [25], used within the UK for classification of materials. This is a leaching method (using water) that is implemented throughout Europe based on European Standard [26] that is adopted by EU states providing that they meet regulatory thresholds based on hazardous limits set for waste disposal by an EU Council Decision [27].

There are four variations of the EN standard 12457 (e.g., 12457-1, 12457-2, *etc.*): Part 1 is a single stage batch test 2.0 L/kg water-waste ratio; part 2 is a single stage batch test at 10.0 l/kg ratio; part 3 is a two-stage batch test using both 2.0 L/kg and 8.0 L/kg, and part 4 is a single batch test with 10.0 L/kg ratio but with particle sizes below 10 mm (1–3 specify particle size below 4 mm). Within the EU, individual countries have adopted this procedure (Table 1) as a routine testing protocol. It can be seen that the UK has no set routine procedure, however WAC testing is used with a corresponding guidance document [28]. The WAC specifies leaching limit values defined by the Acceptance Leaching Test (BS EN 12457 [29]) based upon parameters that include total solid concentration, moisture content, leached content, pH and Eh that are used to assess risk to the environment and ultimately establishes the potential for their disposal, removal or recovery.

**Table 1:** Use of EU standard “EN 12457” in European countries, including variations in approaches to use and implementation [30].

Country	Regulation of The Use of Waste Aggregate?	Criteria on Total Content?	Criteria on Leaching Content?	Type of Leaching Test
Austria	Guidelines	Yes	Yes	EN 12457-4 (L/S = 10.0 l/kg)
Czech republic	Based on landfill legislation	Yes	Yes	EN 12457-4 (L/S = 10.0 l/kg)
Denmark	Yes	Yes	Yes	EN 12457-1
Finland	Yes	Yes	Yes	EN 12457-3 and CEN/TS 14405
France	Yes	Yes	Yes	EN 12457-2 and 4
Germany	Yes (new reg in preparation)	Yes	Yes	EN 12457-2 (& new leg. DIN 19528)
Italy	Yes	No	Yes	EN 12457-2
Spain	Yes—by region	No	Yes	EN 12457-4 (& Din 38414-s4)
United Kingdom	Case by case guidance	No	No	Variable—no set routine

Criteria on: total content or leaching content refers to whether each country includes data of the different chemical measurements.

Although many countries within the EU apply EN 12457 [29] or a similar, or more stringent screening processes, there are still problems with the testing scheme itself. A key issue not currently addressed is the stability of PTEs once leached out, and whether precipitation can occur [12].

The chemistry of the contaminants of interest can be difficult to evaluate with leaching tests due to the compounds or complexes in which they are hosted. Although no consideration is given to speciation of the contaminants of interest in WAC and similar leaching tests, it is known that this can affect solubility under specific conditions [31]. Consequently, this may affect rates dissolution and precipitation and, where there is a high organic matter content, rates of adsorption and desorption. Speciation may also influence the mobility, bioavailability and ultimately the potential for toxic effects within the wider environment [12].

Despite its limitations, WAC testing dictates landfill disposal routes for wastes (hazardous, non-hazard and inert) that not only come with high costs but are becoming more restrictive for producers as threshold limits reduce [32]. Developing a tool to improve industrial management is required to be able to over come these ever growing constraints.

### 1.3. Significance of Speciation

Prediction of the fate of the PTEs on disposal can be established by studying the environmental conditions surrounding landfills and within waste deposits in order to establish how trace elements that are incorporated in the waste combine and interact with phases in the surrounding soil [33]. A further key factor in determining the fate of PTEs is their chemical form, which combines with the environmental factors to influence their mobility or stability. Chemical form and distribution of an element may both be refered to as its “speciation”. IUPAC (International Union of Pure and Applied Chemistry) defines “speciation” in three ways:

- The specific form of an element, such as, its electronic or oxidation state, complexation, molecular structure or isotopic composition.
- The distribution of an element amongst defined chemical species in a system.
- Analytical procedures for identifying and/or measuring the quantities of one or more individual chemical species in a sample.

These three definitions, when combined, provide a complete speciation of elements [23].

In steel wastes, the main constituents that cause “hazardous” classification are PTEs (Pb, Zn) and alkali metals (e.g., K), due to their concentration and potential environmental impact. The environmental impacts of these constituents depend strongly on their mineralogical and chemical form, as these dictate their mobility and bioavailability [34]. The ability to speciate in solid matrices analytically is often a difficult and complex process. However, the benefit from this knowledge concerning mobility and bioavailability of the elements of interest may provide better understanding and routes to the minimization of environmental impact [17].

If a fundamental understanding of the chemical and physical characteristics of PTEs found within wastes arising from the steel industry can be established, then a more informed and appropriate approach can be implemented for their removal, stabilization and/or re-use [35]. For example, in baghouse dusts collected from electric arc furnaces the main zinc phase present is known to be zinc ferrite (Zn_x_Fe_3–x_O_4_), but hydrometallurgical cleaning techniques struggle to digest this compound [36]. With the use of synchrotron analytical technology both amorphous and crystalline zinc structures have been identified in blast furnace sludge (BFS). These include Zn phyllosilicates, Zn sulphide minerals (e.g., sphalerite, würtzite), KZn-ferrocyanide phases, hydrozincite and tetrahedrally coordinated adsorbed Zn, all having different stabilities and key reactivity characteristics [11]. These different Zn hosts are specific to individual waste types because the steel production process is inherently variable; the semi-continuous batch process varies from site to site due to different operational configurations and specialist production demands.

The ability to apply a single characterisation technique to a solid sample and obtain full speciation may be desirable when attempting to understand the fate of PTEs from wastes,, however it is not always practical, economic or even possible. Nevertheless, relevant information can be inferred by establishing characteristic chemical traits of the waste under simulated environmental conditions. One such approach is sequential extraction.

#### Sequential Extraction (SE) as a Technique for Waste Characterisation

It is widely known that the application of strong acid leaching can be used to determine the total content of PTEs in solid phases but results in an overestimation for environmental exposure. Varying acid or other reagents sequentially by increasing strength or type (*i.e.*, acidity or dissolution ability), can result in the successive solubilisation phases and release of associated elements from operationally defined fractions in the samples [37]. This approach is known as sequential extraction (SE) [38,39,40,41], originally applied to soils and sediments [12,42]. This technique can aid speciation by understanding the reaction behaviour of individual solid phases (fractions) as their presence may be inferred from their dissolved products in leachates extracted. SE also has an advantage over WAC water leaching as it provides a more realistic prediction of elemental mobility within the environment as extraction reagents are applied in to mimic environmental processes e.g., acid rain [43].

In the following sections we provide a detailed overview of SE methodologies and thus identify the current status of SE and its potential for application to successfully characterise different forms of steel waste. We conclude that this technique shows promising potential for its application within the waste management process.

## 2. Wastes from the Steel Industry

Wastes from steel production contain a range of potentially toxic elements (PTEs). The levels of risk that these present to environmental and human health are mitigated through processes such as dilution, dispersion, oxidation, degradation or sequestration into soils and sediments [44]. The sources of waste streams in the steel making process determine and the nature of the phases making up the bulk composition and hence their content of harmful materials.

### Nature of Wastes

Steel is an alloy of iron and carbon, with other constituents such as lead, zinc, manganese, phosphorus, nickel, silicon, sulphur and chromium present at significant concentrations as impurities (from feedstock) or that have been added at various stages of the process [45]. Its production begins with a sinter plant, where the raw feed (e.g., limestone, coke feed, olivine, hematite, magnetite and recycled materials from the blast furnace (BF) and basic oxygen furnace (BOF) are mixed and heated to high temperatures as a pre-treatment process [46,47], resulting in the production of sinter. This is then heated in the BF to temperatures above 1000 °C with iron ore, and the addition of coke and limestone. Here, the coke is burnt to produce carbon monoxide, which is a key reducing agent, that in turn reduces the iron ore oxide as it travels up the shaft to iron [48]. Finally the molten pig iron from the BF and ferrous scrap (up to 30%), are refined into steel by injecting a jet stream of high-purity oxygen (HPO) through the hot metal [49].

These high-temperature processes create significant quantities of sludge, slag, and dust that may generate toxic and hazardous by-products as well as valuable metals [50]. Such waste types are generated at different production points with different physical characteristics: Sludge is a semi-solid material consisting of fine particulate material (0.02–0.3 mm) collected during the purifying of BF gases, and flue dusts with the addition of water [51]. Dusts have the finest particles (>200 μm) and are collected by extraction and gas cleaning systems [52]. Slag is a heterogeneous, porous material generated as a by-product of the impurities that are solidified during the BF process [43]. The concentrations of these constituents can vary depending on the type of waste and its intended use (Table 2: “bulk composition” refers to constituents contributing significant portion of the bulk mass).

**Table 2:** Illustrative bulk composition of steel wastes with example of variations observed in the literature [43,53].

Dust [54]	CaO (1–5%)	SiO_2_ (6%–9%)	MgO (<2%)	Al_2_O_3_ (2%–6%)	P_2_O_5_ n/a	TiO_2_ n/a	Fe (48%–52%)	K_2_O (0.1%–2%)	Na_2_O n/a	S n/a	C (29%–34%)
Slag [43,55]	CaO (30%–60%)	SiO_2_ (10%–35%)	MgO (1%–6%)	Al_2_O_3_ (0.5%–4%)	P_2_O_5_ (0.5%–15%)	TiO_2_ (0.4%–2%)	Fe (7%–80%)	K_2_O n/a	Na_2_O n/a	S (<0.1%)	
Sludge [54,56,57]	CaO (6%–14%)	SiO_2_ (2%–10%)	MgO (<0.1%)	Al_2_O_3_ (<0.1%)	P_2_O_5_ (<0.01%)	TiO_2_ n/a	Fe (8%–66%)	K_2_O n/a	Na_2_O n/a	S (<1.5%)	C (7%–40%)

NB: Fe refers to various Fe forms FeO and Fe_2_O_3_.

By identifying the key components of the different wastes, predictions can be made about the chemical structures present and how it affects reactivity in the environment. The main amorphous component of slags is glass, whilst crystalline constituents present are Fe, Ca, and Mg oxides, hydroxides, silicates and carbonates, with elemental Fe and quartz [43]. When exposed to simulated environmental conditions (water vapour saturated air current passed over and through slag) the formation of carbonate phases occurs that reduces the leaching of alkaline earth elements e.g., Ca, Mg and Cr(VI and III) [58]. Phases in BF wastes are composed predominately of silica and alumina derived from mineral impurities in original iron ore, along with Ca and Mg oxides from added fluxes [59]. This combined with its finely particulate structure and exposed internal surface area can result in chemical reactions taking place and can alter porosity and permeability e.g., by corrosion or precipitation, which has been shown to significantly decrease the leaching of metals [51].

Elements of particular interest with respect to the environmental and industrial processing impact (Table 3) include potassium, lead and zinc, which are all known to be toxic to some degree depending on their chemical form. However, they are not typically assessed in regulatory analysis [60]. These constituents are problematic due to their potential toxicity, but they may also be “poisonous” to the production process [61], which can degrade the potential for recycling in the production cycle.

**Table 3:** Typical composition of key constituents of steel wastes (mass %).

	Sinter Dust [62]	BOF Sludge [61]	BF Sludge (Gas Treatment) [61]	BF Sludge (Dry) [63]	BF Sludge (from Landfill) [57]
Fe	43–50	48–70	7–35	21–32	5.7–27.5
C	2.9–6.12	0.7–4.6	15–47	1.0–3.2	7–40
Pb	0.09–5.98	0.04–0.14	0.8–2	0.3–1.2	0.1–2
Zn	0.03–0.34	0.2–4.1	1–10	1.0–3.2	1.5–8.6
K	3–9.07	n/a	0.08–0.36	n/a	0.1–1.7
Ca	7.55–7.83	3.0–17	3.5–18	n/a	3.5–13.5

## 3. Sequential Extraction

Sequential extraction is a common analytical method used to identify elements associated with solid phases in environmental media on the basis of their reactivity with specific solutions. It has been applied to the analysis of sediments since the early 1980s [64,65,66,67,68,69], and since then has been applied to soils [70,71,72] and waste materials [73,74].

This method establishes the environmental reactivity of PTEs by progressively applying a series of chemical reagents that are selected to release elements of interest associated with the respective phases, resulting in the fractionation of the element distribution in samples [75,76]. The extracts are separated from the sample and analysed directly, usually by atomic spectroscopy techniques for the elements of interest. The fraction of an element associated with each extraction step is compared to the “total” sample content obtained by a bulk chemical analysis of the sample. The sum of component fractions should equal an independently determined total value [77]. This technique could aid speciation by characterising steel wastes in accordance to fractions (phase associations).

Direct methods for more routine determination of solid-state speciation e.g., SEM, XRD, FTIR, are potentially of great value in characterizing waste materials, they can demonstrate intermediate to short scale variability, but are generally of insufficient sensitivity for environmental trace analysis or require multiple units of fairly specialized equipment to add sufficient new information for useful characterisation [78].

### 3.1. Obstacles to the Application of SEP

Sequential extraction is limited by the fractionation processes displacing chemical elements from a range of compounds resulting in chemical alteration of the matrix and potentially falsely indicating the presence of particular chemical phases in the sample. Furthermore this approach is not considered a fully quantitative method for the following reasons: the analyte may be re-distributed among solid phases during each extraction step rather than being extracted with the aqueous leachate; the reagents may not be sufficiently selective for the target phase; extraction may be inefficient and hence incomplete. In addition, new solid phases may be precipitated from the leachate [12]. The latter is also known to be a problem with other aqueous extraction methods such as WAC testing and is therefore common across all extraction protocols. The verification of “success” of SE protocols can be through the agreement of summed fractions with independent totals. These methods are also susceptible to particle loss during sample handling, particularly the loss of fine sample material when separating each fraction during centrifuging, decanting and washing. Typically only the leachate is analysed due to the destructive nature to the solid material, whereas when used together with solid-state analysis e.g., X-ray absorption spectroscopy sample integrity can remain in tack and the additional solid phase analysis can provide a more reliable outcome, for example predicting long term efficiency of *in situ* soil treatments [33].

The aggregation state of the sample may also cause inefficiencies in extraction. For example, mineral or organic coatings on sample particles, or other textural characteristics of multiphase particles, may prevent access of the reagent to some constituents. This could be overcome by crushing or grinding the sample. However, this can expose fresh mineral faces/surfaces with a different leaching response and metal release compared to matrices in real environmental conditions [12].

### 3.2. Defining Fractions

SE attempts to extract metals of interest by targeting pre-defined solid-state fractions of the sample having particular physico-chemical characteristics. In other words, it is a process that fractionates the sample according to its solubility or reactivity. Fractionation in this sense may be defined as: “the process of classification of an analyte or a group of analytes from a specific sample according to physical (e.g., size, solubility) or chemical (e.g., bonding, reactivity) properties” [23]. This approach does not, strictly, “speciate” samples, but it identifies the associations of the different fractions with known elemental and chemical species. Importantly, this fractionation approach to sample characterisation attempts to emulate natural fractionation processes that may mobilise toxic species in the environment, so it has operational value in the identification of the environmental impacts of waste systems.

In evaluating metal fractionation in steel wastes, we may draw upon decades of research on metal mobility and fractionation in ground and surface water [79], soils and sediments [80] and sewage sludge [81]. In contrast, relatively little information is available on the leaching and extraction methods that have been applied to industrial waste materials. Overall, however, we may conclude that the success of fractionation is affected by the the strength of the metal-ligand bonds associated with each fraction [82], as well as variations in pH, redox state, organic content and other environmental factors [83,84].

**Figure 2:**
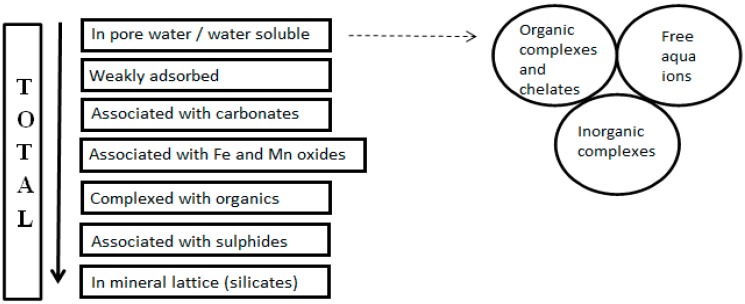
Chemical forms of metals in solid phases (Modified from [84]).

The reagents used for extraction are selected to target metals that may be essential major components of major solid phases (e.g., major framework-forming cations in crystalline solids) and/or bound into, or adsorbed onto solid phases by particular modes of bonding. The resulting range of SE protocols addresses typical groups of extraction targets with a varying number of steps, and the use of different nomenclature to label the associated phases. Commonly defined fractions are the *exchangeable fraction*, which targets weakly adsorbed chemical species and can be referred to as water- or acid-soluble, this can also include free aqua ions, inorganic and organic complexes; the *carbonate fraction*, which can be included with, or separated from, the exchangeable fraction; the *reducible fraction*, which can be referred to as the fraction associated with Fe and Mn oxides; the *organically bound* (oxidisable) fraction, and the *residual* (silicate) fraction [12,84,85,86,87]. Such operationally defined fractions can be difficult to equate with “metals bound to specific phases” when the procedural steps are not indicated [12]; this is a critical omission in many studies. Previous research has shown that similarly labeled fractions may be derived from different experimental procedures for extraction [88], which can provide very different results [71,89,90]. Therefore the expectation of similar results is only valid when more detail is disclosed in the description of methodology, providing a more in-depth explanation of the operationally-defined steps (see Figure 2).

For all fractions, typical extraction reagents are presented in Table 4, and relate to studies of to soil/sediment matrices. Fractions are identified and measured according to the elements leached from the solid phase into solution. Different extraction reagents have been used (Table 4, Table 5 and Table 6), to target similar solid phases, however the justification of their use by linking to specific reaction mechanisms is not common in SE development. Very few studies justify reagent composition on the basis of evidenced solid phase reaction.

**Table 4:** Modifications of BCR sequential extraction procedure (1993–2008).

Sample Type	Fraction 1 (Exchangeable, Water and Acid Soluble)	Fraction 2 (Reducible (Fe and Mn—Oxyhydroxides)	Fraction 3 (Oxidisable—Organic Matter and Sulphides)		Fraction 4 (Residual—Silicate Bound)
	Reagents	Reagents	Reagents		Reagents
Soils and sediments [41]	0.11 moL·L^−1^ acetic acid 2 h [91]	0.1 moL·L^−1^ hydroxyl-ammonium chloride pH 2, 4 h [91]	hydrogen peroxide followed by 1.0 moL·L^−1^ ammonium acetate at pH 2		*aqua regia*
Sewage sludge [92] Sediment [68]	0.11 moL·L^−1^ acetic acid	0.5 moL·L^−1^ hydroxyl - ammonium chloride pH 1.5	hydrogen peroxide followed by 1.0 moL·L^−1^ ammonium acetate at pH 2		*aqua regia*
N/A [12]	0.11 moL·L^−1^ acetic acid	0.5 moL·L^−1^ hydroxyl-ammonium chloride	H_2_O_2_, 1.0 moL·L^−1^ CH_3_COONH_4_		Aqua regia
Sewage sludge [93]	40 mL 0.11 M Acetic acid	40 mL 0.10 M hydroxylamine hydrochloride (pH 2 HNO_3_)	10 mL 8.8 M H_2_O_2_ AND 1 M Acetic acid		HF
Solid wastes [74]	0.11 moL·L^−1^ Acetic acid	0.1 moL·L^−1^ hydroxylamine hydrochloride (pH 2 HNO_3_)	8.8 M H_2_O_2_	1 moL·L^−1^ ammonium acetate (adjusted pH HNO_3_)	Perchloric acid—hydrofluoric acid, hydrochloric acid.
Municipal sewage sludge [81]	0.11 moL·L^−1^ and CH_3_COOH	0.5 moL·L^−1^ hydroxyl-ammonium chloride pH 1.5	H_2_O_2_, 1.0 moL·L^−1^ and CH_3_COONH_4_ at pH 2		7 mL HNO_3_ + 2 mL HF + 1 mL HClO_4_
Marine Sediments [94]	0.11 M Acetic acid	0.10 moL·L ^−1^ hydroxylamine hydrochloride (pH 2 HNO_3_)	30% H_2_O_2_ pH 2 (HNO_3_) AND 1 M Acetic acid pH 2 (HNO_3_)		Hot HNO_3_ conc.
Marine Sediments [95]	20 mL 0.11 M Acetic acid	20 mL 0.10M hydroxylamine hydrochloride (pH 1.5 by addition of 2 moL·L^−1^ HNO_3_)	5 mL 8.8 M H_2_O_2_ AND 1 M Acetic acid		HNO_3_ and HF
Marine Sediments [69]	Acetic acid 0.11 moL·L ^−1^ pH 2.85	Hydroxyl ammonium chloride (NH_2_OH.HCl 0.1 moL·L^−1^) pH 2	30% H_2_O_2_ (8.8 moL·L^−1^ ) , followed by CH_3_COONH_4_ (1 moL·L^−1^) pH 2		Mix HNO_3_ (2 mL) and H_2_O_2_ (2 mL) + HF (0.5 mL)

**Table 5:** Modifications of Tessier sequential extraction procedure (1979–2005).

Sample Type	Fraction 1	Fraction 2	Fraction 3	Fraction 4				Fraction 5	
	Reagents	Reagents	Reagents	Reagents				Reagents	
Tessier (original) fluvial sediments [64]	8 mL 1M MgCl_2_, pH 7.0, 1 h	8 mL 1M NaOAc, acetic acid to pH 5, 6 h	20 mL 0.04 M NH_2_OH-HCl in 25% (v/v) HOAc, 6 h	3 mL 0.02 M HNO_3_, 5 mL 30% H_2_O_2_ (pH 2 with HNO_3_), 65 °C, 3 h	3.2 M NH_4_OAc in 20% (v/v) HNO_3_		3 mL 30% H_2_O_2,_ pH 2 HSO		HF-HClO_4_
N/A. [12]	1 M MgCl_2_ (pH 7.0)	1 M NaOAc, acetic acid to pH 2	0.04 M NH_2_OH-HCl in 25% (v/v) HOAc, 96 °C	HNO_3_/H_2_O_2_ (85° C)	Then		3.2 moL·L^−1^ NH_4_OAc in 20% (v/v) HNO_3_		HF-HClO_4_
MSW [96]	1 M CH_3_COOH/CH_3_COONa pH 5, 5 h	NH_2_OH-HCl 0.1 M, 40 mL	K_4_P_2_O_7_ 0.1 M, 20 mL, pH 9.5, 20 °C, 24 h	NH_2_OH-HCl 0.04 M in CH_3_COOH 25%, 20 mL, 60 °C, 6 h				HNO_3_-HCl Conc 12 h 20 °C, 3 h at 105 °C	
Soil [91]	1 M MgCl_2_, pH 7.0	1 M NaOAc/acetic acid pH 5	0.04 M NH_2_OH-HCl in 25% (v/v) HOAc	0.02M HNO_3_ in 30% H_2_O_2_ pH 2		3.2 M NH_4_OAc in 20% (v/v) HNO_3_			
Marine sediment [94]	1 M MgCl_2_, pH 7.0, 1 h	1 M NaOAc/acetic acid, 5 h	0.04 M NH_2_OH-HCl in 25% (v/v) HOAc 6 h—96 °C	30% H_2_O_2_ pH 2 (85 °C), 5 h		3.2 moL·L^−1^ NH_4_OAc in 20% (v/v) HNO_3_, 0.5 h		Hot HF-HClO_4_	
Soil [97]	8 mL 0.5 M MgCl_2_, pH 7.0, 20 min	8 mL 1 M NaOAc, 5 h	0.04 M NH_2_OH-HCl in 25% (v/v) HOAc, 6 h—96 °C	3 mL 0.02 M HNO_3_ and 5 mL 30% H_2_O_2_ Heated 2 h 5 mL 3.2 M NH_4_Oac, 0.5 h				4 mL conc. HNO_3_, and 2 mL HCl Microwave	
Sediments [98]	1 M MgCl_2_, pH 7	1 M NaOAc/acetic acid, pH 5	0.04 M NH_2_OH-HCl in 25% (v/v) HOAc	30% H_2_O_2_, pH 2 with HNO_3_				HF + HClO_4_ + HNO_3_	
MSW [99]	1 M NH_4_Ac, pH 7	1 M NaAc, pH 5	0.1 M NH_2_OH-HCl pH 2, 12 h	40 mL 0.1 M oxalate buffer, pH 3				30% H_2_O_2_ (pH 3), 1 M NH_4_Ac (pH 7), 12 h	
Soils (Galán) [100]	1 M NH_4_OAc, pH 5	n/a	0.4 M NH_2_OH-HCl in CH_3_OOH 25%	0.2 M HNO_3_, 30% H_2_O_2_, pH 2 30% H_2_O_2_ then another H_2_O_2_				HF, HNO_3_, HCl, 10:3:1	

MSW—Municipal solid waste.

**Table 6:** Sequential extraction application to industrial wastes with characteristics similar to steel making wastes (key observations identified, recoveries that are not quoted were either not stated, or based on the residual fraction being the assumed difference of the pseudo total and the preceding steps, *i.e.*, residual fraction not experimentally measured).

Sample		Elements	Remarks
4 fractions [101] 1990	Mining wastes	Cu, Cd, Zn, Pb	Low leachability in water was observed with majority of metals found in residual fraction. Zn showed high levels in the acid soluble, reducible and residual fractions. Cu was found in the oxidizable fraction and Pb in the reducible fraction.
Adapted Tessier SEP [102] 1995	Municipal solid waste incinerator ash	As, Cd, Cu, Hg, Pb, S, Zn	The pH of the resulting leachate is the greatest factor governing the concentration of metals in solution. This out ways concentrations in the ash.
SE based on Tessier [103] 1996	Scale, sludge	As, S, Cu, Cr, Zn, Pb	Both scale and sludge consisted mostly of oxides of Si, Al and Fe. The sequential extraction showed that As, Cu and Zn were leachable under extreme conditions.
5 fractions [104] 1996	Landfill liners	Pb, Ni, Cd,	A new method: combined SE–sorption isotherm analysis. SE data indicated Pb and Ni were principally in the acid soluble fraction, and Cd was in the exchangeable fraction.
Sequential extraction [105] 1998	Dust	Pb	SE revealed Pb in exchangeable fraction was less than 7% and mildly acidic steps for the bulk dusts collected. The finer particle size factions from these areas of smelter showed higher percentages of exchangeable lead.
5-step [106] 2008	BOF Flying dust	Zn	Reference materials were used to show Zn species ZnCl_2_ and ZnSO_4_ extracted from the exchangeable fraction, ZnCO_3_ in carbonate fraction, and ZnS from the reduced fractions. Complications with selectivity to ZnO as was released during the second and third extraction step. So cant distinguish ZnCO_3_ from ZnO.
BCR [107] 2008	Sludge	Cd, Cu, Cr, Ni, Pb, Zn	Different sludges shows BCR recovery between 80%–100%. SE a higher degree of mineralisation and stabilisation can occur by its lowered metal bioavailability – predicted as a result of the associated to the oxidisable and residual fractions.
Revised BCR [108] 2013	Slag	Al, As, Ba, Be, Co, Cr, Cu, Fe, Hg, Mn, Mo, Ni, Pb, Sb, SE, S, V, Zn	Showed significant recoveries 88%–109%.
Tessier [109] 2013	Bottom Ash	Cu, Cd, and Zn	The results showed that the fractionation of Cu, Zn and Cd varied among the different size particles, and was greatly dependent on the intrinsic property of the metal species and their transfer behavior in the furnace.
BCR [110] 2015	BF Slag	Al, Ba, Co, Cr, Cu, Fe, Mn, Mo, Ni, Pb, S, V, Zn	Showed difficulties regarding Zn recovery during step 1 and Cu recovery during step 2
5-step [42] 2015	BF Sludge	Hg	Specifically optimised Hg focused SEP that was proven successful for BFS with recoveries 73%–114% despite being optimised for soils.

#### 3.2.1. Exchangeable Fraction

The exchangeable fraction, sometimes referred to as the acid-soluble/water soluble fraction, is envisaged to comprise water-soluble elements as well as ion (H^+^) exchangeable and carbonate bound metals. This fraction represents the natural environmental effects of acidic rainwater percolation [111]. These metals are also considered to be mobile and can be used to quantify the short term availability for leaching or uptake by plants [112]. They are removed from the solid phases (raw steel wastes) by electrostatic interactions and the changing ionic composition of water. This allows metals sorbed on exposed sediment surfaces to be removed easily by adsorption-desorption processes [100], thus ultimately by ion exchange.

Mobility and ease of extraction into the environment can depend on solubility. Metal hydroxide minerals have a low solubility under high pH conditions in water due to hydroxyl ion activity being inversely related to pH. As pH decreases, solubility increases and more metals are liberated into solution [113]. In the aqueous environment the free sites are easily occupied my neutral salts [114].

In soils when heavily contaminated, many metals at high concentrations will form precipitates with oxides, hydroxides and carbonates, especially at higher pH [85].

The solid-phase hosts of his fraction can act as a major adsorbent in industrial sludge, providing a high bioavailability because any change in pH may affect the processes of adsorption-desorption and ultimately the mobility of metals [107]. The cation exchange capacity (CEC) is also an influencing factor, where silicate minerals can provide cation exchange (CE) sites for adsorption/desorption of the metals [115]. Typically, steel wastes contain 30%–35% silicates [60] and approximately 30% for typical soils [116]; in soils these tend to predominately consist of phyllosilicates with a high CEC however steel wastes have a mixture with anhydrous and orthosillicates with a lower CEC. However, silicates in soils, when combined with their organic matter content, give a correspondingly higher joint cation exchange capacity [117] and therefore a greater affinity to retain metals.

In most soils the exchangeable fraction accounts for less than 2% of total metals with the exceptions of microelements K, Ca, and Mn [99].

As previously mentioned this fraction can be separated into a further fraction; the carbonate bound fraction. Carbonation by absorption of atmospheric CO_2_ and addition during any waste processing steps (such as by the addition of lime) results in significant carbonate abundance in many waste streams. This can lead to redistribution of element associations, but is dependent on physical properties. For example, it has been observed [107] that metal partitioning within sewage sludge is strongly influenced by stabilization treatments (whether by a chemical or mechanical approach), and when introduced to soil most of the metals will associate or complex with the carbonates present, despite the presence of other reactive phases. Larger particles are associated with higher contaminant concentrations and are more likely to be encased by carbonate coatings, hence reducing the efficiency of reagents and consequently are not redistributed between the sequential extraction steps [35].

#### 3.2.2. Reducible Fraction

The reducible (Fe-Mn oxides) fraction represents metals bound to iron and manganese (and sometimes aluminium) oxides that would be released if the solid matrix were susceptible to anoxic (reducing) conditions [100]. It has been shown that the oxides often act like a cement between particles and form coatings on mineral surfaces particularly in soils, whilst fine particles can be present due to a combination of precipitation, adsorption, surface complex formation and ion exchange [20]. The extraction of the metals bound to this phase, whether as Fe-oxides, Mn-oxides or both, is dependent on the efficiency of the selected reagents used previously. For example it is possible that carbonates have not been completely dissolved in preceding steps. It is also possible that the content of Fe and Mn hydroxides is low and release would not be detected [111].

The process of adsorption/desorption is strongly dependent on pH and the availability of particulate surfaces to bind to. Cd and Zn for example have adsorption edges at higher pH’s compared to Fe and Cu and consequently are more mobile and widely dispersed [81,85,118]. This can occur as a combination of the precipitation, adsorption, surface complex formation and ion exchange [119]. Particle size varies greatly in steel wastes (see Table 7). This can can effect available surface areas, adsorption processes and 3 ultimately bioavailability [120]).

**Table 7:** Typical particle size distribution of industrial waste materials.

Fly Ash	Flue Dust	BOF Sludge	BF Sludge
Size from 0.5 to 300 µm	0.075–0.250 mm dominated in the flue dust [121]	From less than 5 μm to as large as 1 mm [122]	Up to 1.5 mm fine-grained 1–10 μm coarser part 10–100 μm, where 90% of particles are below 50 μm [53]
0.1–500 μm with majority between 20–60 μm [123]	P50 of 41.468 µm P10 17.57 µm, P90 was 83.6 µm [124]	Average particle size: Fine fraction ~37 μm, Coarse fraction ~210 μm [125]	Percentage distribution [126]: 2–5 mm = 13.65 1.25–2 mm = 32.53 0.8–1.25 mm = 15.56 0.2–0.8 mm = 20.2810.40 0.08–0.2 mm = 10.20 <0.08mm = 7.78
2 μm–10 μm [127]		<0.7–43 μm range with main fraction falling in 14–22 μm fraction [128]	

Metals bound into oxide phases are considered stable. However, when incorporated with other compounds (e.g., by adsorption, desorption or ion exchange, *etc.*) in the environment they can be slowly released over time, or influenced by redox (reduction-oxidation) conditions [85]. Furthermore major cations such as Mg^2+^ and Ca^2+^ compete for sorption sites with other metals [113], which will be a major factor in BF wastes [60] as this can result in the liberation of its PTEs in the environment.

#### 3.2.3. Oxidisable Fraction

The oxidisable fraction consists of organic- or sulphide-bound elements and metals. They may be complexed or peptised by natural organic substances. They can bind with functional groups such as carboxyl, phenol, alcohol, carbonyl and methoxyl [129]. In soils and sediments, these pollutants are assumed to remain within the solid matrix and are mobilized after a significant period of time, usually by the decomposition of organic matter, or are liberated when exposed to oxidising conditions [20,107]. The extracts obtained during this extraction step when dealing with steel wastes are typically metals bound to sulphides [130].

The most commonly used reagent for the extraction of metals in organic phases is hydrogen peroxide with ammonium acetate. However HNO_3_ with HCl has also been used to dissolve sulphides as it provides enhanced selectivity. However, this extraction mixture is aggressive towards silicates [131], and makes it less selective and unapplicable for preceeding steps e.g., exchangeable or reducible fraction.

The organic-bound fraction released in the oxidisable fraction SE step is considered not to be bioavailable due to the fact that it is associated with stable high molecular weight humic substances that release small amounts of metals slowly [132]. Metals can be adsorbed onto organic matter and mineral surfaces in inorganic and organic forms and transported throughout the environment [133], which is relevant to steel wastes when they are released to the wider environment.

#### 3.2.4. Residual Fraction

The residual fraction is where metals have the strongest associations with crystalline structures of primary and secondary minerals, *i.e.*, they are the most difficult to extract. This fraction is relevant in the assessment of long-term risk of PTEs [20].

The elements associated with this fraction are of little direct risk to the environment as the strong acids (e.g., concentrated HCl and HNO_3_,or both in *aqua regia*) used to leach in the extraction do not reproduce conditions that are ulikely to occur naturally [100]. These digestion techniques have potential to dissolve only a small amount of the silicate matrices (up to 20% content) and therefore naming this fraction the “silicate” phase is misleading, even if stronger digestion methods are used and partial solubility of silicate phases does occur.

The association of metal content with this fraction is defined by some authors as the difference between the pseudo-total concentration (strong acids—aqua regia) and the sum of the preceding fractions [134]. This may not be reliable due to summation error and the residual phase should strictly relate to complete dissolution (e.g., HF). However, the pseudototal is truly a maximum for many assessment purposes because achieving such extreme chemical conditions in the environment is highly unlikely. It should be recognized that in waste materials in particular, there may be a significant but highly inert portion of the elements of interest in this phase e.g., spinels.

### 3.3. Contribution of SE to Sample Compositional Assessment

The different fractions defined above can provide data relating to the reactivity and mobility (Table 7) of samples that can aid prediction of the fate PTEs within the environment. Identifying the characteristic traits of these fractions can assist indirectly in deriving likely speciation of elements in these waste materials.

## 4. The Development of the Sequential Extraction Approach

SE has been developed over the past 3–4 decades starting with a sequential extraction method originated by Tessier, Bison and Campbell in 1979 [64]. This is a 5-step procedure that separates element solid associations into exchangeable, acid soluble, reducible, oxidisable and residual fractions (Table 8). This basic scheme has been subjected to numerous modifications in order to increase reproducibility and efficiency of extraction [132,135]. This has been achieved by using different experimental conditions and extracting reagents [136,137,138,139].

**Table 8:** Relative mobility and availability of trace metals (modified from David, 1995 and Tessier 1979 [64,84].

Metal species and association	Description	Mobility
Exchangeable (dissolved) cations	fraction affected by ionic composition, pH, sorption and desorption processes	High. Changes in major cationic composition (e.g., estuarine environment) may cause a release due to ion exchange
Metals associated with Fe-Mn oxides (Reducible)	consists of metals attached to iron and manganese oxides and which are unstable under anoxic conditions	Medium. Changes in redox conditions may cause a release but some metals precipitate if sulfide mineral present is insoluble
Metals associated with organic matter (Oxidisable)	can be released when the organic matter is degraded leading to release of soluble metals under oxidizing conditions	Medium/High. With time, decomposition/oxidation of organic matter occurs
Metals associated with sulfide minerals	The sulfide minerals are a class of minerals containing sulfide (S^2−^) as the major anion.	Strongly dependent on environmental conditions. Under oxygen-rich conditions, oxidation of sulfide minerals leads to release of metals
Metals fixed in crystalline phase (Residual)	Predominantly primary and secondary minerals, which may hold metals within their structure	Low. Only available after weathering or decomposition

As previously mentioned the development of SEs’ application developed a number of operational problems such as non-selectivity, redistribution of trace elements, re-adsorption, precipitation, extractant sufficiency and problems related to physical handling during sample preparation and drying techniques [111].

The original scheme was developed for the assessment of the potential impact of sediment bound PTEs on water quality [12]. However, the rapid extension to soils [140] and municipal solid wastes [99] resulted in significant modification of extraction steps and reagents which, in turn, made it difficult to compare data when evaluating results. The need for standardization resulted in research commissioned by the Community Bureau of Reference of the Commission of the European Communities (now called the European Community (EC) Standards Measurement and Testing Programme), which led to a harmonized three-stage, sequential extraction protocol known as “BCR” targeting the acid soluble, reducible and oxidisable fractions [12,72].

The BCR method *was* validated using a sediment reference material (BCR-701) and sewage sludge amended soil (CRM-483) providing certified and indicative extractractable concentrations for Cd, Cr, Cu, Ni, Pb and Zn [92,128]. This has been widely accepted as a reference standard [94,141,142,143,144,145] despite some shortcomings in the sequential extraction steps [146,147]. It is important to compare these methods: The biggest difference is the separation of extractable metal fractions, so that Tessier’s first two steps equate to the total metal concentration in step one of BCR’s method. This overlap is not distinct due to procedural differences including contact time, temperature and the solid to extractant volume ratio [132]. As with Tessier’s method, the BCR SEP has been modified over time to optimise SE efficiency and success in fractionating a range of sample types (Table 4).

Unlike the temperature, which is frequently quoted in SEP reports (Table 4 and Table 5), because it is considered a key operational parameter for reactivity; the extraction time or experimental length is not necessarily specified. Table 4 shows a variation of between 16–50 h and Table 4 only identifies the time period for only half of one method. The Galan procedure which incorporates both Tessier and BCR steps had a 14.5 h total extraction time and showed increased accuracy compared to the separate procedures [100]. The Geological Society of Canada (GCS) provides thorough detail of time requirements with a 5 step SEP totaling 21.2 h [148].

Insufficient contact time between the solid and the extracting reagent can be detrimental in ensuring complete extraction or the highest efficiency [149]. This can be considered a key factor when identifying the best procedural approach for industrial managers. It is worth noting that time constraints are not necessarily included in procedural descriptions, neither are the total separation time between steps. This means that the full resource implications of SEP application are not fully disseminated.

The continuous development of these procedures means no consistent extraction reagent list for the various components in sediment, soils and waste materials has been defined. This is exacerbated by matrix effects of different samples and the reagent “recipe” is strongly dependent on the waste composition. For example steel wastes are very base-rich compared to soils (see Table 2 and Table 3), so there is a larger acid-buffering capacity during extraction [150].

### Key Factors Affecting the Effectiveness of SE

The reason for the continuous modifications has been fuelled by the need to optimise the method for each different sample matrix. Factors that need to be considered include: the sequence of steps, specific matrix effects (such as cross-contamination), pH buffering, re-adsorption, precipitation, as well as physical characteristics (e.g., coatings, inclusions) of the various solid fractions [151].

**Figure 3:**
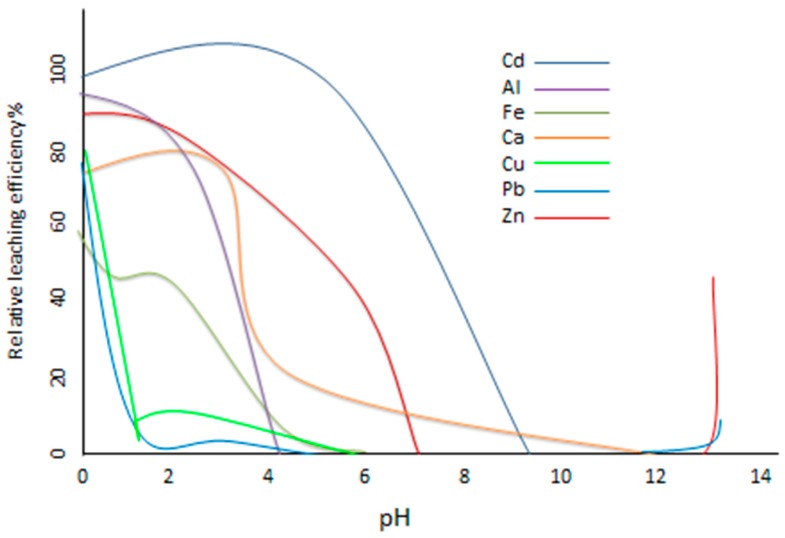
Relative leaching efficiency (%) of elements as a function of the final pH for MSWI (municipal solid waste incinerator) fly ash [155].

A European framework project on the “Harmonisation of leaching/extraction tests” when comparing SE application to different solid materials (e.g., soils, sediments, mining wastes, fly ash, *etc.*), found that despite variation in matrix properties, the extraction processes were more consistent than would be anticipated [152], suggesting that matrix variation is not critical to method development in SE. The pH and buffering capacity of solids can greatly effect extraction and ultimately leaching efficiency (Figure 3). This has been considered in the context of the high pH of the water-soluble fraction, which leads to the formation of hydroxides that can result in the precipitation of metals. Although there is empirical evidence for part of the interaction with the leaching solution it is difficult to specify the phases involved. Previous research from Herck *et al*. [153] used a computer modelling program (Visual MINTEQ) to establish which minerals precipitate in terms of leaching efficiency as a function of pH (Figure 3). It can be seen that the higher the pH the lower the leached element, although above pH 12 Pb and Zn start to leach out again [154].

Further investigation by comparison of modelled data and SE of fly ash was carried out to confirm necessary conditions to optimise extractions, with general agreement that it is successful [150,153,155]. This research highlighted fundamental information that needs to be considered when selecting a SE procedure. Zinc for example at pH 6, has about 31% of its total concentration present in solution, and 26% is precipitated as smithsonite and 20% as ZnO.SiO_2_. At pH 4.5, zinc solubility decreases due to the precipitation of zinc silicate; at pH 8 different zinc minerals occur and no more zinc remains in solution and by pH > 13.5, zinc solubility increases again, due to the formation of hydroxide complexes [153].

Its application for modeling the speciation of steel slags has also been explored [156], showing unexpectedly that the key factors controlling the leaching of Cr and Ba are the presence of the mineral phases barite (BaSO_4_ and hashemite (Ba(S,Cr)O_4_). It was also observed that the presence of Na made pH measurements unreliable and therefore elemental predictions could be inaccurate [155].

It can also be noted the considerable variation in pH of soil compared to fly ash and steel slag (Table 9) could suggest that using sequential extraction of fly ash as a reference matrix would be more relevant than that of data form soil extractions as they both have high alkaline properties that will consequently dictate the materials reactivity [157].

**Table 9:** Variation of pH in different sample matrices [157].

Variable	Soil	Steel Slag	BF Sludge [54]	Fly Ash
pH	3.9	12.5	9.88	13.1

The majority of sequential extraction investigations are applied to sludge, sludge-amended soils, sediments and analysing the waste by-products that have leached or been composted into amended soils, whereas very few studies deal with direct applications to waste materials [96]. The literature on the application of SEP to more diverse waste materials is limited. The following section considers the major differences in material behaviour.

## 5. Application of SE to Different Wastes

The applications of the Tessier and BCR schemes to steel waste analysis across the range of different sources are limited. However, waste matrices with similar characteristics have been observed including sewage sludge, fly ash, bottom ash and BF slag, all of which should exhibit similar effects due to similar characteristics such as particle size and elemental composition. These waste materials are often alkaline and contain solid phases able to adsorb and immobilize metals. Fly ash and steel slag, for example, have similar compositions that include calcium oxides, magnesium oxide, iron oxide, calcium silicates, calcium aluminates, and other silicates and oxides [157].

The appropriate reagents and experimental parameters are determined by assessing extraction success by its recovery (based on total recovered vs pseudo total digest) and based on the ability of its procedural steps to remove the desired analytes from specific phases [12].

Although the marjoity of examples throughout this review are soil and sediment focused there are examples of SEPs that have been successfully applied to different solid types Table 6.

Although BCR is still widely used for sequential extraction (Table 4), it has been reported to show miss-classification for Zn during the first step and Cu in the second step when compared to certified materials for slags [110], sediments [158,159] and mining waste [17].

As previously noted, the lack of procedural detail in many descriptions and the wide range shown for those described more fully *i.e.*, procedural steps; such as heating, contact time, *etc.*, has been proven to be fundamental to the ability to successfully apply SEP [12]. Furthermore the percentage recoveries are often not stated and given experience of a number of intercomparison studies can make data difficult compare.

To be able to compare SE procedures applied to different samples other factors such as pH (Table 9) and particle size (Table 7). The reactivity of the sample matrix plays a vital role in the success of extraction, *i.e.*, smaller particle size results in faster reactions.

BOF and BF sludge show that the particle size distribution can exceed 1.0 mm (Table 7) whereas flue dust and fly ash have a similar particle size that would suggest that these samples types could react in a similar manner. Therefore research showing characterisation (90–110) of fly ash could be a good starting point when deciding upon a SEP to adopt for steel flue dust characterisation. The physical and chemical properties of MSWI fly ash, such as particle size and chemical composition are known to influence the decomposition of metal host phases obtained by sequential extraction [153,160,161,162,163].

The comparison of the effectiveness of a SEP can be assessed by application in parallel to different industrial wastes, ensuring the same extraction reagents and operational steps are adhered to. A 7-step SEP was applied to extract metal fractions from mine wastes targeting seven operationally-defined phases: water-soluble, ion-exchangeable, carbonate, amorphous Fe-oxide, crystalline Fe-oxide, sulfide, and silicate [164]. The success of extraction was found to depend on particle size; a trend of increased concentration in Fe, Pb, Zn and Cu as the particle size of waste decreased particularly with water-soluble, Fe-MnOx_am_ and sulphide fractions, and the inverse with the FeOx_cryst_ attributed to the lack of dissolution of larger pyrite crystals [164].

This approach was further applied to operationally define metal associations in electric arc furnace dust (EAFD) [165] through the opportunity to provide more “complete” fractionation. Metals bound within spinel-type phases (residual fraction) were distinguished as a very stable component through the need for strong acid digestion. The six other steps are subject to sequential release of metals according to the conditions of solubility, pH and Eh.

SE can offer a higher resolution by the application of larger numbers of steps, also providing additional information, particularly when steel wastes are concerned. For example, the 7-step SE distinguishes between amorphous and crystalline Fe oxides/hydroxides. Logically, amorphous Fe will be more reactive in soil components as they represent a transition state between un-weathered parent materials and well-crystallized secondary soils minerals. These are also key forms of Fe found in steel waste; hematite (Fe_2_O_3_) and goethite (FeOOH) tend to occur as amorphous coatings on particles whereas transformation to crystalized Fe results in an irreversible hardening and altering the relative availability of associated elements [116].

## 6. Conclusions

As waste management regulations within Europe become more stringent and the option to landfill wastes becomes an increasingly unlikely future option, a critical need exists for industrial operators to assess the sustainability of the current production processes. Using SE to speciate the chemical composition of solids wastes provided higher resolution information to allow alternative uses or solutions for hazard reduction of wastes, whether it is stabilization for reclassification or extraction of valuable PTEs, which feeds back directly to production costs.

As no mandatory SEP exists, the options available for waste characterisation must be adopted on the basis of waste material properties. The consistency of approach is most critical. Although the BCR approach has been widely applied to a range of different sample types and a limited number of reference materials are available, the operational approach varies widely and in the case of wastes from the steel industry provides misleading data specifically with zinc, which is a critical constituent of wastes from all production processes. Tessier’s procedure whilst having been historically the most applied has least methodological verification and typically has been applied to soils and sediments for a small array of elements.

SEP is an analytical tool that can provide a great detail of additional information relating to the environmental reactivity of solid samples. As an approach, it is far from routine, particularly in relation to speed of data collection. This is also true of other methods and may not be an issue where management plans are being developed. However, part of this relates to the poorly reported procedural data which suggests a variation in length of assay from a few hours to 2+ days, not including separation time between steps. Its value lies in its efficiency to characterise waste types that can then be assessed for future treatment/stabilisation options. Characteristics that are found to be similar in other sample matrices, such as metal constituents, particle size and pH provide comparator opportunities for reference points. For example, soils have typically a larger maximum particle size (and wider size distribution range) and a significantly lower pH, provide less chemically buffered conditions. Fly ash (which has been widely subject to a range of optimised SEP schemes) has similar characteristics to materials from steel processing e.g., pH steel slag 12.5 and Sludge 9.8, fly ash pH 13.1 and may offer an opportunity of harmonization across a range of problem solid wastes. Steel waste management can benefit from the use of more complex SEPs as they offer a much higher resolution of the compositional detail. In particular the delineation of iron phases, which also has implications for more sustainable fly ash and mining waste management. Ultimately we note that the prospect for improved characterisation of steel wastes using SE is a viable step to advance industrial management procedures.
